# Concerns about cognitive performance at chance level

**DOI:** 10.1038/s41598-021-93953-8

**Published:** 2021-07-30

**Authors:** Leonardo Jost

**Affiliations:** Faculty of Human Sciences, University of Regensburg, Regensburg, Germany

**Keywords:** Psychology, Human behaviour

**arising from**: A. J. Toth and M. J. Campbell; *Scientific Reports* https://doi.org/10.1038/s41598-019-56041-6 (2019).

## Introduction

Psychometric mental rotation tests have shown some of the largest gender differences in cognitive psychology. In psychometric mental rotation tests, two rotated stimuli out of four possible stimuli have to be identified in each trial. Toth and Campbell^1^ investigated this using pupillometry and fixation metrics to analyze cognitive effort and strategies. The performance data shows that on average, participants identified only 17.353 out of 40 stimuli correctly. As always half of the stimuli had to be selected and all participants completed all trials, this is even below chance level. Similarly, in the more common scoring method, participants identified both rotated stimuli in 2.765 out of 20 tasks, which is again below chance level of 3.33/20. I argue that this suggests a possible misunderstanding of the task by participants but at least calls into question the interpretation of the results.

Despite the authors’ effort to ensure that participants understood the task, I feel two main questions still remain: (1) how is it possible that participants performed this poorly and (2) can we meaningfully analyze performance at chance level? But I will also outline, were the authors have plausibly explained that errors are unlikely.

While I agree that both the pupillometric data and the reaction time suggest cognitive effort by the participants this does not suggest that the effort was invested in mental rotation. It is possible that participants misunderstood the task and performed a different cognitive task or even malingered. This is somewhat unlikely, as the authors have also shared the instructions to the task in the supplementary material and they seem understandable.

Another possibility is that answers were recorded incorrectly and the accuracy data contains errors. The data suggests a systematic difference between data from mirrored and structural distractors, which in turn suggests that answers were not recorded randomly. If an error occurred here, it was likely systematic. As the authors have laid out their rigor concerning the data collection and analysis, this should not be a possible explanation.

Of course, it is also possible that the participants were just that bad at mental rotation but this is extremely improbable given the sample size of 70 participants. That result in itself would be astonishing as in no mental rotation study have adult participants ever performed even close to this poorly. Large scale studies report scores more than 2.5 times higher even for the lower scoring females (n = 1765 and n = 1218)^2,3^. The participants in this study were also psychology and sport science undergraduate students, who are common participants in mental rotation studies. The possibly most comparable cohort of Campbell et al.^4^ also showed performance (in a chronometric test) comparable to other mental rotation studies. The lowest scores in mental rotation tests are reported for elementary school children^5,6^. Yet the lowest score achieved by 8-year-old girls of 3.87/24 is still better than the comparable score here of 2.765/20. Furthermore, the children only had 6 min time for 24 tasks, which typically produces even lower scores^2^. Hoyek et al.^5^ even conclude that the task was probably too complex to analyze the gender differences in question.

The data also suggests worse performance on structural distractors. These are typically easier, as no mental rotation is required to identify them^7^. Thus, the difference should even be enhanced for participants, which are bad at mental rotation. One plausible reason is outlined by the authors in the limitations section, as these tasks were not practiced.

If participants were completely guessing or performing a different cognitive task, their behavior would clearly not be related to their performance. However, even if they were in fact this bad at mental rotation, the question remains, if performance at chance level can be meaningfully analyzed. While it is not the intended research question of the study and the problem possibly arose through no fault of the authors, the data requires the discussion.

I believe performance at chance level cannot be meaningfully analyzed. We cannot infer from the data in any way, if and what cognitive effort lead to finding the correct answer. This is supported by many common research practices: wrong answers are typically excluded from analyses (except accuracy data) because it is not clear for which reasons errors occur. At chance level, it is also not clear for which reasons correct answers occur and we would have to exclude all data. Moreover, many studies invest effort to identify and exclude guessers from analysis. Guessing is typically identified as performance at or below chance level. This may not be the optimal procedure but if only few guessers are excluded this should not majorly impact analyses. If however half of the participants were to be excluded by such a procedure and as random answers lead to a binomial distribution of correct answers, the logical conclusion would be that all or almost all participants were guessing.

Guessing in this context does not mean that participants saw the task and immediately guessed without investing cognitive effort. Imagine a multiple choice task in quantum physics that far exceeds our knowledge. Reading and trying to understand possible answers would require both time and cognitive effort but in the end, our answers should be indistinguishable from randomness. Inferring strategies from tasks, which we managed to answer correctly, would be meaningless and trying to find patterns in this data would only mean finding patterns in samples of randomness. Similarly, trying to identify why some participants performed better than others would be equally meaningless.

Consequently, I believe the discussion of the connection between eye measurements and performance in the present study is fruitless. What can be investigated on the other hand is the overall gaze pattern independent of performance, the analysis of pupil diameter as an overall marker of cognitive effort, and possible sex differences. However, these must not be linked to the mental rotation task itself as they could be measurements of attempting an unsolvable task, and subsequently overall stress and even frustration. As the distinction between foils is arbitrary outside the context of the task, one possible conclusion could be that both males and females show similar fixation patterns and a comparable stress response when faced with a task that is too complex to solve.

Another conclusion might be that it is indeed possible that the mental rotation task used in this study is too complex for many participants. In this case, it should be investigated which details of the methodology influence the task difficulty in this way. The resulting similar performance of males and females should however not serve as contradictory evidence to sex differences in other mental rotation studies.

## References

[1] Toth AJ, Campbell MJ (2019). Investigating sex differences, cognitive effort, strategy, and performance on a computerised version of the mental rotations test via eye tracking. Sci. Rep..

[2] Peters M (2005). Sex differences and the factor of time in solving Vandenberg and Kuse mental rotation problems. Brain Cogn..

[3] Krüger JK, Suchan B (2016). You should be the specialist! Weak mental rotation performance in aviation security screeners—Reduced performance level in aviation security with no gender effect. Front. Psychol..

[4] Campbell MJ, Toth AJ, Brady N (2018). Illuminating sex differences in mental rotation using pupillometry. Biol. Psychol..

[5] Hoyek N, Collet C, Fargier P, Guillot A (2012). The use of the vandenberg and Kuse mental rotation test in children. J. Individ. Differ..

[6] Titze C, Jansen P, Heil M (2010). Mental rotation performance and the effect of gender in fourth graders and adults. Eur. J. Dev. Psychol..

[7] Voyer D, Hou J (2006). Type of items and the magnitude of gender differences on the mental rotations test. Can. J. Exp. Psychol..

